# Paper-Based Airborne Bacteria Collection and DNA Extraction Kit

**DOI:** 10.3390/bios11100375

**Published:** 2021-10-07

**Authors:** Youngung Seok, Joonseok Lee, Min-Gon Kim

**Affiliations:** 1 Department of Mechanical Engineering and Applied Mechanics, School of Engineering and Applied Science, University of Pennsylvania, 216 Towne Building, 220 S. 33rd Street, Philadelphia, PA 19104, USA; yuseok@seas.upenn.edu; 2 Department of Chemistry, Hanyang University, Seoul 04763, Korea; joonseoklee@hanyang.ac.kr; 3 Department of Chemistry, School of Physics and Chemistry, Gwangju Institute of Science and Technology (GIST), 123 Cheomdan-gwagiro, Gwangju 61005, Korea

**Keywords:** airborne pathogens, DNA extraction, bacteria detection, environment, air monitoring, lateral flow strip, bio-aerosol

## Abstract

The critical risk from airborne infectious diseases, bio-weapons, and harmful bacteria is currently the highest it has ever been in human history. The requirement for monitoring airborne pathogens has gradually increased to defend against bioterrorism or prevent pandemics, especially via simple and low-cost platforms which can be applied in resource-limited settings. Here, we developed a paper-based airborne bacteria collection and DNA extraction kit suitable for simple application with minimal instruments. Airborne sample collection and DNA extraction for PCR analysis were integrated in the paper kit. We created an easy-to-use paper-based air monitoring system using 3D printing technology combined with an air pump. The operation time of the entire process, comprising air sampling, bacterial cell lysis, purification and concentration of DNA, and elution of the DNA analyte, was within 20 min. All the investigations and optimum settings were tested in a custom-designed closed cabinet system. In the fabricated cabinet system, the paper kit operated effectively at a temperature of 25–35 °C and 30–70% relative humidity for air containing 10–10^6^ CFU *Staphylococcus aureus*. This paper kit could be applied for simple, rapid, and cost-effective airborne pathogen monitoring.

## 1. Introduction

Infections from airborne pathogens have become an increasingly serious risk for global public health. For the past two decades, infectious diseases have spread worldwide, resulting in numerous victims and a significant social burden [1,2,3,4,5]. These include the viruses severe acute respiratory syndrome-associated coronavirus (SARS-CoV) in 2003 [1], a novel influenza A (H1N1) in 2009 [2], and Middle East respiratory syndrome coronavirus (MERS-CoV) in 2015 [3] that are transmitted through air and respiratory routes. As one of the most critical cases, SARS-CoV-2, which causes COVID-19, emerged in late 2019 and is an ongoing pandemic with unparalleled historical global impacts [4,5]. As seen with these cases, the current global population is at a much higher risk than before due to its increased size and advanced transportation technologies. This may also allow biochemical weapon attacks using airborne microorganisms to take place, such as anthrax, chicken pox, or tuberculosis [6]. To prevent the spread of diseases or defend against bioterrorism, rapid and low-cost detection techniques are required which can be used in various regions with harsh conditions, such as rural and mountainous areas or places with limited resources.

Most airborne pathogens are existing types of bio-aerosols [7], and airborne pathogens are generally detected via aerosol collection and analysis of collected microorganisms. Due to occasional extremely low level concentrations and much larger volumes compared to other types of samples, air sampling has been a bottleneck for the diagnosis of airborne pathogens [8,9]. Currently, many types of air samplers for aerosol collection have been developed [10,11,12,13,14,15]. To measure bacteria, colony counting on solid cell culture media is one of the most traditional and widely used methods [16]. Airborne bacterial pathogens are collected by settlement [10] or specific sampling skills [12,14], and the number of bacteria is measured.

Colony counting is a basic, effective, and reliable technique; however, colony formation, microscopic techniques, and lab setup with skilled personnel require a long time (>24 h) [17]. Alternatively, aerosols and airborne pathogens can be collected on filter paper [18,19]. Bio-aerosols in the air can be captured in the functional paper material during air pump filtration. In other types, induced inertial/gravitational movements in the sampler send aerosols to a pre-loaded solution [9], with preservation of microorganisms. These techniques could be applied for the monitoring of airborne pathogens combined with appropriate biosensors. However, some aerosol collection techniques require heavy instruments, long sampling times, or additional complex settings. The availability of rapid, accurate, and easy-to-use techniques with effective aerosol analysis is still a technical issue [7,20].

Point-of-care testing, which is simple, robust, rapid, low-cost, and sensitive and provides a specific diagnosis, has dramatically progressed, with medical application trends focusing on prevention and early diagnosis [21,22,23,24,25]. Regarding a simple and low-cost method for mass testing, a paper-based analysis device has been accepted as being one of the most promising diagnostic platforms [22]. To resolve unmet needs in the detection of airborne pathogens, several studies have used a paper platform [26,27,28]. Airborne pathogen collection on paper material could also be an effective alternative for a simple and portable system which is easily combustible without any risk of contamination. These previous studies collected airborne pathogens which can be effectively treated and analyzed through an additional paper connection or biocompatible downstream reactions, such as FTA membranes, paper-based analytical devices, lateral flow immunoassays, or molecular diagnostics.

Herein, we developed a paper-based airborne bacteria collection and DNA extraction kit. We collected bacteria with an air pump and then integrated an appropriate porous material in the handheld DNA extraction strip developed in our previous study [29]. Purified and concentrated DNA on the binding pad was usable for direct PCR analysis. The 3D printing case improved the connection between the air pump and DNA extraction strip to enable extensive application of the paper kit. To create a pathogen-contained air environment, we fabricated a closed cabinet system which produces aerosols with bacteria at different temperatures and humidity. In the cabinet system, we tested a collection of airborne bacteria on the paper kit for various working conditions using a cultured solution of *Staphylococcus aureus (S. aureus)* bacteria.

## 2. Materials and Methods

### 2.1. Materials 

A conjugate glass pad was purchased from Ahlstrom (Helsinki, Finland). GF/F-grade and GF/C-grade glass filters (GFs) were purchased from Whatman (Maidstone, Kent, UK). A vivid plasma separation GF was purchased from Pall Co. (Port Washington, NY, USA). Polycarbonate, nylon, and quartz fiber were purchased from Advantec (Dublin, CA, USA). A cellulose pad was purchased from Vericel Co. (Ann Arbor, MI, USA). ELISA sealing tape was purchased from Excel Scientific (Victroville, CA, USA). Oligonucleotide primers were purchased from Genotech (Daejeon, Korea). Reagents for real-time PCR analysis were purchased from Enzynomics (Daejeon, Korea). NaOH, sodium dodecyl sulfate (SDS), and Triton X-100 were purchased from Sigma-Aldrich (St. Louis, MO, USA). Molecular-grade water was purchased from Invitrogen (Carlsbad, CA, USA).

### 2.2. 3D Printing

The case was designed using SolidWorks™ (Dassualt Systems) computer-aided design (CAD) software and exported as an STL file for 3D printing on a Formlabs (Sommerville, MA) Form 3 low-force stereolithographic desktop 3D printer in clear photopolymer resin. Hand-drawn, 3D images of the printing case for the paper kit are shown in Figure 2a–c. After printing, printing supports were removed, and the parts were washed with isopropyl alcohol in the Form Wash (Formlabs) unit for 15 min, followed by post-curing for 15 min with 405 nm UV light at 60 °C in the Form Cure unit. A photograph of the 3D-printed case is shown in Figure 2d.

### 2.3. Paper Kit Assembly

**Figure 1:**
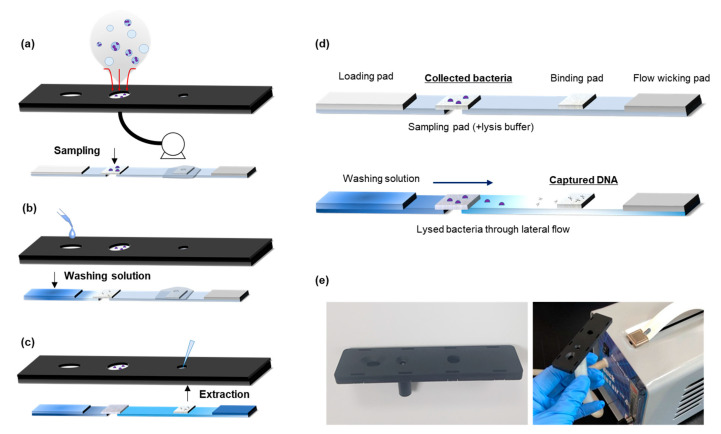
Workflow of the paper kit: (**a**) aerosol sample collection using air pump, (**b**) loading of washing solution at hole in the case, (**c**) DNA extraction through lateral flow in the strip, (**d**) schematic of aerosol collection and DNA extraction process, and (**e**) photographs of the paper kit with 3D-printed case and air pump.

**Figure 2:**
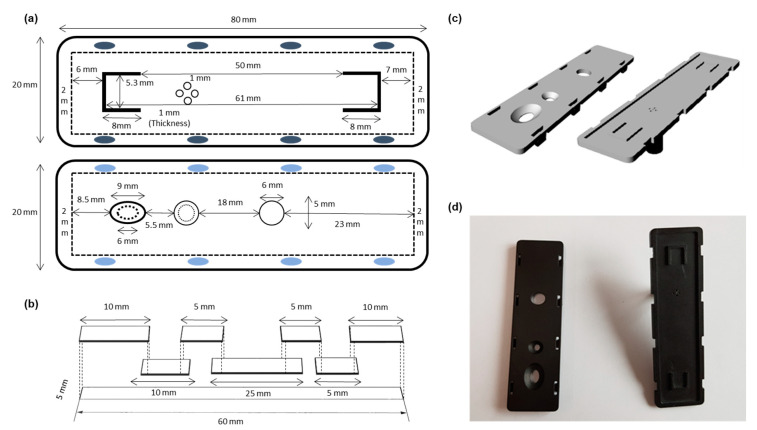
3D printing of airborne bacteria collection kit. (**a**) Hand drawing of the 3D printing chip, (**b**) simple drawing of the paper strip with assembly line in the case, (**c**) CAD image, and (**d**) photograph of printed case.

The designed paper-based airborne bacteria collection kit is composed of transfer, loading, sample, binding, and flow wicking pads. The overall structure is schematically shown in Figure 1d. The vivid plasma separation GF was the transfer pad, whereas the GF/C-grade, conjugate, and GF/F-grade glass pads were used to fabricate the loading, sample, and binding pads, respectively. The absorbent pad was placed at the top of the strip structure to induce fluidic flow in the paper device by acting as a flow wicking pad. The transfer pad was cut to an appropriate length and attached on backing card (PJIGO Seoul, South Korea) following the overall design. Loading (10 mm), sample (5 mm), and wicking (15 mm) pads were attached to the remaining part of the backing card after being cut to uniform length. Subsequently, all parts of the paper system were attached, the assembled backing card was cut into uniform 5 mm strips, and each strip was placed into a 3D-printed case. Size information and the overlap in each attachment are shown in Figure 2b. The binding pad (5 × 5 mm) was attached at the required position using ELISA sealing tape with a 1.5 mm-diameter center hole. Finally, 20 μL of lysis buffer (200 mM NaOH with 1% SDS) was loaded on the sample pad, which was fully dried.

### 2.4. Operation of Paper Kit

The paper kit operates in three steps: (1) sampling, (2) washing, and (3) extraction of DNA, as shown in Figure 1. Air samples with airborne bacteria were collected in the sampling section of the case and combined with the air pump (LinEair 40 LPM sampling pump, 230VAC, A.P. BUCK Inc., Orlando, FL, USA). The air sample was passed through the sampling pad during the operation of the air pump, and airborne pathogens simultaneously attached to the porous surface. Subsequently, 75 μL of the washing buffer (15% (*v*/*v*) isopropyl alcohol) was injected into the buffer hole at the bottom of the case [29]. After 3 min, 2 μL of distilled water (D.W.) was injected and transported up the hole of the elution pad. The aliquot of this eluted solution was analyzed by downstream analysis and quantitative real-time PCR.

### 2.5. Staphylococcus Aureus Bacteria Culture

*Staphylococcus aureus* (*S. aureus*, ATCC 23235) was cultured in Tryptic Soy Broth (Hach, CO, USA) at 37 °C for 18 h inside an incubator shaken at 200 rpm. To count the colony-forming units (CFUs), grown cells were serially diluted 10-fold in fresh Tryptic Soy Broth medium and plated on Tryptic Soy Agar (Hach) plates, which were subsequently incubated for 18 h at 37 °C. After overnight incubation, each colony dot was counted once, and the number of grown *S. aureus* cells was recorded. Finally, the number-counted *S. aureus* culture was diluted in phosphate buffer saline (PBS) solution.

### 2.6. Quantification of S. aureus by Real-Time PCR (qPCR)

The primer for real-time PCR (qPCR) was designed by targeting the *S. aureus* 16S ribosomal RNA gene from the NCBI database. The DNA sequence of the forward primer was 5′-GCACATCTTGACGGTACCTAATC-3′, and the sequence of the reverse primer was 5′-CGCGCTTTACGCCCAATAA-3′. A commercial real-time PCR kit, 2× qPCR ROX mixture, was used for qPCR. The reaction samples (20 μL) contained primers (100 nM each), 1× commercial qPCR mixture, and 2 μL of the sample containing template DNA. The thermal profile used was: 95 °C held for 10 min, 40 cycles of 95 °C for 30 s, 63 °C for 30 s, and 72 °C for 35 s. 

The corresponding CFUs of *S. aureus* were calculated using the 16s rDNA region and the designed PCR primer. All real-time PCR reactions were run on a CFX96 real-time PCR instrument (Bio-Rad Laboratories, Hercules, CA, USA), and the quantity of analyzed DNA was determined using CFX96 software using the relationship between CFU and Cq values (Figure S2).

### 2.7. Closed Cabinet System

#### 2.7.1. Fabrication of Closed Cabinet System

The closed cabinet was custom fabricated with acrylic plates. A schematic of the cabinet system is shown in Figure S1. The cabinet operated as a completely closed system and could be handled inside using a built-in glove, in the same fashion as a glove box in a chemical lab. The cabinet consisted of a body, nebulizer, and compressed air line to produce the aerosol, vacuum pump, and control box with a thermometer and relative humidity meter connected to a heater and humidifier. A pass box was also attached to the cabinet body to move experimental material in and out during the experiment.

#### 2.7.2. Pressure, Temperature, and Humidity Control

Pressure, temperature, and humidity can be controlled automatically using a control box. At the beginning of the experiment, we set the range of each factor, e.g., ±3 mm H_2_O pressure, 25.0–25.5 °C temperature, and 50–55% humidity. When the temperature in the cabinet was lower than the intended setting, the inside heater was set to the correct temperature. When humidity in the cabinet was lower than the desired setting, the inside humidifier provided more vapor. When humidity was higher, compressed dried air was injected into the cabinet, and the humidity was reduced. A vacuum pump was also operated to maintain the pressure in the cabinet system during the aerosol loading experiment. 

#### 2.7.3. Production of *S. aureus* Aerosol

Cultured *S. aureus* was diluted in 10 mM PBS to the desired concentration for each test. The diluted bacteria solution was combined at the air inlet port on the top of the cabinet (Figure S1b). A connected aerosol generator (3-jet Collison nebulizer, CN24, Mesa Labs, Butler, NJ, USA) was nebulized into a myriad of droplet aerosols under an air flow of 5 L/min. The produced aerosols were directly injected into the chamber, simulating the airborne pathogen environment conditions. 

#### 2.7.4. Ultraviolet Exposure Remediation

The cabinet had a 260 nm ultraviolet (UV) lamp inside which could be turned on/off using the control box. The UV lamp in the cabinet was used to sterilize the tested bacterial aerosol. After 10 min of UV radiation, most bacteria and viruses are destroyed [30]. The total elimination of loaded *S. aureus* bacteria in the cabinet was confirmed by real-time PCR and cell growth results in the solid broth (Figure S1c,d). The chamber was cleaned with UV treatment between each nebulization event.

### 2.8. Operation of Impactor Sampler

One impactor sampler, which works by air pump-accelerated collision of the bio-aerosol with the solid broth, was used as a representative technique of the cultured bacterial colony counting method. The Microflow alpha (90-C kit, Aquaria Srl, MI, Italy) is a portable impactor sampler which is convenient and suitable for air quality testing in clean facilities. We used the impactor sampler following the manual instructions at a 120 L/min flow rate for 15 min with Tryptic Soy Agar (Hach, CO, USA) medium on a 90 mm plate.

## 3. Results and Discussion

### 3.1. Airborne Bacteria Collection and DNA Extraction

Figure 1 describes airborne bacteria collection and DNA extraction using the paper kit. The workflow of the paper kit from airborne pathogen collection to obtaining the extracted DNA is illustrated in Figure 1a–c. The air sample was filtered through the porous structure of the sampling pad using the air pump, and bacterial cells accumulated on the pad (Figure S4). Lateral flow separation through the porous materials in the paper kit was a rapid DNA extraction process [31]. The porous surface of the paper material has a strong binding affinity with DNA molecules based on capillary fluidics and the entanglement effect [32]. The principle of DNA extraction in the strip is shown in Figure 1d. The collected bacteria were lysed when they mixed with the dried lysis buffer on the sampling pad, and DNA molecules were released in the bacterial cell. The DNA was transported to the binding pad by lateral flow of the washing solution and was captured on the binding pad due to the difference in binding affinity within other porous materials [29]. Other contaminants such as cell debris, proteins, or dust particles in the air were removed from the wicking pad or filtered by the prior structure of the binding pad.

### 3.2. Assembly of Paper-Based Airborne Bacteria Monitoring Kit

For effective operation of the system, the paper kit requires a fitted case that ensures easy operation, including combining the air pump and solution loading. The case of the paper kit, which supports the whole system (Figure 2a), was printed using a 3D printer. The end user of the paper kit can easily recognize the functions of each part and how to apply the system for airborne pathogen monitoring. A 3D illustration of the case and real photographic image are also displayed in Figure 2c,d. The sampling pad and dried lysis buffer materials were tested to optimize the kit. In the closed cabinet system, 2 mL of *S. aureus* solution (5.0 × 10^4^ CFU/mL) was injected into the cabinet by inlet airflow and analyzed under various conditions (Figure S3). Conjugate glass pad material and a basic condition lysis buffer with SDS were chosen for the paper-based airborne bacteria monitoring kit.

### 3.3. Operation Factors of Air Sampling

**Figure 3:**
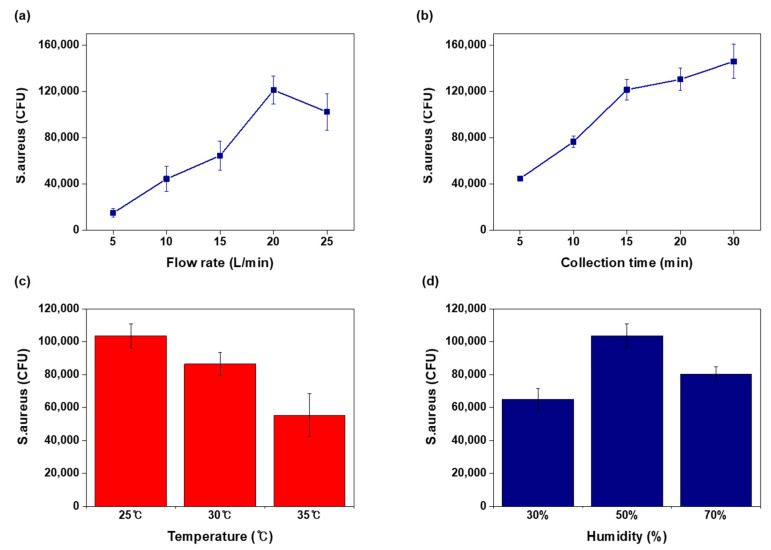
Investigation of various variables for airborne bacteria collection kit. All CFU values were measured by qPCR curves, *N* = 3. (**a**) Flow rate of air pump testing, (**b**) bacteria collection results for pump operation time, (**c**) bacteria collection results by different temperatures, and (**d**) collection results by humidity.

The closed cabinet system should maintain a constant temperature and humidity during aerosol analysis [33]. In the cabinet system, we collected and analyzed airborne pathogens under the same conditions for the entire experiment. Various factors for airborne bacteria collection of the paper kit were tested under the same temperature (25 °C), humidity (50%), and number of bacteria (10^6^ CFU of *S. aureus*) conditions. The first variable in the airborne bacteria analysis kit was the flow rate of the air pump. When the flow rate is too fast (>25 L/min), bacterial cells may not remain on the paper surface, and a slow rate needs a much longer time to obtain the collection result. Based on the experimental data, 20 L/min is the optimal flow rate of this paper kit (Figure 3a). During the air sampling process, bacterial cells accumulated on the sampling pad during the collection time. We could find the collection time for high efficiency from the DNA analysis results by a 5 min interval (Figure 3b). Temperature and humidity substantially affect the aerosol size [33]; in the test results, the bio-aerosols containing bacteria in the cabinet were also affected by temperature and humidity. Under different temperature and humidity conditions, the number of airborne bacteria from the same nebulizing solution was also changed. The paper kit detected the number of *S. aureus* in the air under a different temperature and humidity, as shown in Figure 3c,d. We verified the reliability of the paper kit when operated at 25–35 °C and 30–70% humidity.

### 3.4. Results of Airborne Bacteria Detection

**Figure 4:**
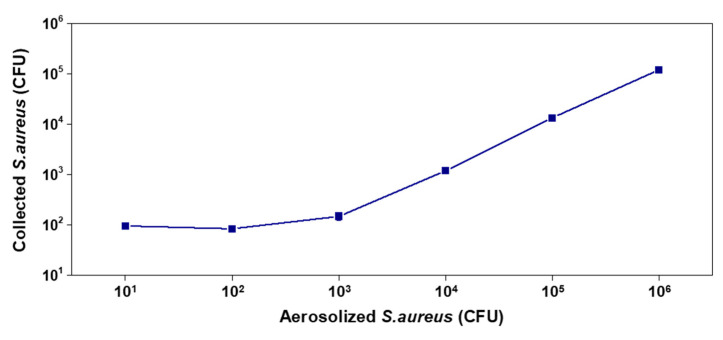
Detection result of collected *S. aureus* using the cabinet system with bacterial aerosol spiking.

The paper-based airborne pathogen monitoring kit was tested at 10–10^6^ CFU of aerosolized *S. aureus* bacteria in Figure 4. At the low bacteria number range (10^1^−10^2^ CFU), most bacteria in the cabinet were collected on the sampling pad. At >10^3^ CFU, the CFU in the kit analysis was lower than the total aerosolized value because not all bacteria were collected in the single kit. The quantity of collected *S. aureus* in the paper was higher when the number of aerosolized bacteria was higher. The relationship between the collected *S. aureus* number and total number of *S. aureus* in the cabinet system was proportional.

Airborne bacteria in indoor air were analyzed using the paper kit at Gwangju, South Korea. We analyzed *S. aureus* in the air on three days in different seasons (Table 1) because *S. aureus* is one of the most common bacterial species in city air. The indoor temperature and humidity were similar on each test day; however, the airborne bacteria concentration differed according to the outdoor environment. The number of bacteria collected with the paper kit indicates the relative concentration of airborne bacteria. We estimated the concentration of *S. aureus* in the indoor air based on the flow rate and operation time of the air pump and compared these values to the colony counting results using an impactor sampler. Each calculation value from the paper kit was overestimated compared to the impactor results by culturing in the cell broth. This was a reasonable result considering the difference between molecular diagnosis (qPCR) and the cell culture technique because dead bacterial cells were also counted in qPCR from the remaining DNA molecules. Following the working procedure of the paper kit, airborne bacteria monitoring was conducted within 20 min, consisting of 15 min of collection and <5 min of DNA extraction. The collection time could be reduced for rapid detection according to the results in the cabinet. This low-cost paper kit can provide valuable information regarding airborne bacteria status in a short time.

## 4. Conclusions

In this study, a paper-based airborne pathogen monitoring kit which can collect bacteria and extract DNA was fabricated. We fabricated an inventive cabinet system which could regulate most factors in the closed setting of the air collection test. Each component of the paper kit and operation factors for air sampling were successfully optimized in the cabinet system, based on a strong binding affinity with DNA molecules [32]. Air collection within 15 min successfully detected airborne bacteria. Artificially contaminated air containing *S. aureus* bacteria in the cabinet was successfully analyzed at various concentrations. Indoor air was also simply analyzed using the paper kit and demonstrated reliable results for the concentration of airborne bacteria. The proposed air monitoring kit is simple, cost-effective, portable, and has mass producible aspects compared to other air sampling techniques. The entire process from sampling to obtaining the DNA analyte took 20 min, which can be reduced when the collection time is reduced. The collected samples and concentrated DNA on the paper kit can undergo PCR analysis after long-term storage during the shipping process [29]. We expect the paper kit could be applied in all locations, including those with limited resources and harsh environments, and become a suitable alternative for air monitoring for disease prevention, bioterrorism defense, air pollution, and other applications.

## Figures and Tables

**Table 1:** Indoor air collection results. Each air collection procedure was conducted in Gwangju, Korea.

		Day 1 (Spring)	Day 2 (Summer)	Day 3 (Autumn)
**Date**		2019.05.15	2019.09.04	2019.11.13
**Outdoor**	**Temperature**	27 °C	27 °C	13 °C
	**Humidity**	48%	90%	65%
**Indoor**	**Temperature**	22 °C	24 °C	22 °C
	**Humidity**	55%	62%	55%
**Paper kit collection**		150 CFU	192 CFU	56 CFU
***S. aureus*** **in the air**	**Paper kit**	500 CFU/m^3^	640 CFU/m^3^	187 CFU/m^3^
	**Colony counting**	36 CFU/m^3^	50 CFU/m^3^	20 CFU/m^3^

## Data Availability

Data is contained within the article or Supplementary Materials.

## Supplementary Materials

The following are available online at https://www.mdpi.com/article/10.3390/bios11100375/s1, Figure S1: Cabinet system for bacterial aerosol experiments, Figure S2: Quantitative real-time PCR (qPCR) analysis, Figure S3, Figure S4: SEM images of sampling pad on the paper kit.
