# Supplementary material for: Paper-Based Airborne Bacteria Collection and DNA Extraction Kit
Source: Biosensors (Basel). 2021 Oct 7;11(10):375. doi: 10.3390/bios11100375 (PMC8533686; doi:10.3390/bios11100375)
Supplement: Supplementary file 1 [file biosensors-11-00375-s001.zip › biosensors-1401820-supplementary.pdf]

biosensors

Supplementary Materials

Paper-Based Airborne Bacteria Collection and DNA Extraction Kit

Youngung Seok [1], Joonseok Lee [2] and Min-Gon Kim [3,*]

1 Department of Mechanical Engineering and Applied Mechanics, School of Engineering and Applied Science, University of Pennsylvania, 216 Towne Building, 220 S. 33rd Street, Philadelphia, PA 19104, USA; yuseok@seas.upenn.edu
2 Department of Chemistry, Hanyang University, Seoul 04763, Korea; joonseoklee@hanyang.ac.kr
3 Department of Chemistry, Gwangju Institute of Science and Technology (GIST), School of Physics and Chemistry, 123 Cheomdan-gwagiro, Gwangju 61005, Korea
* Correspondence: mkim@gist.ac.kr

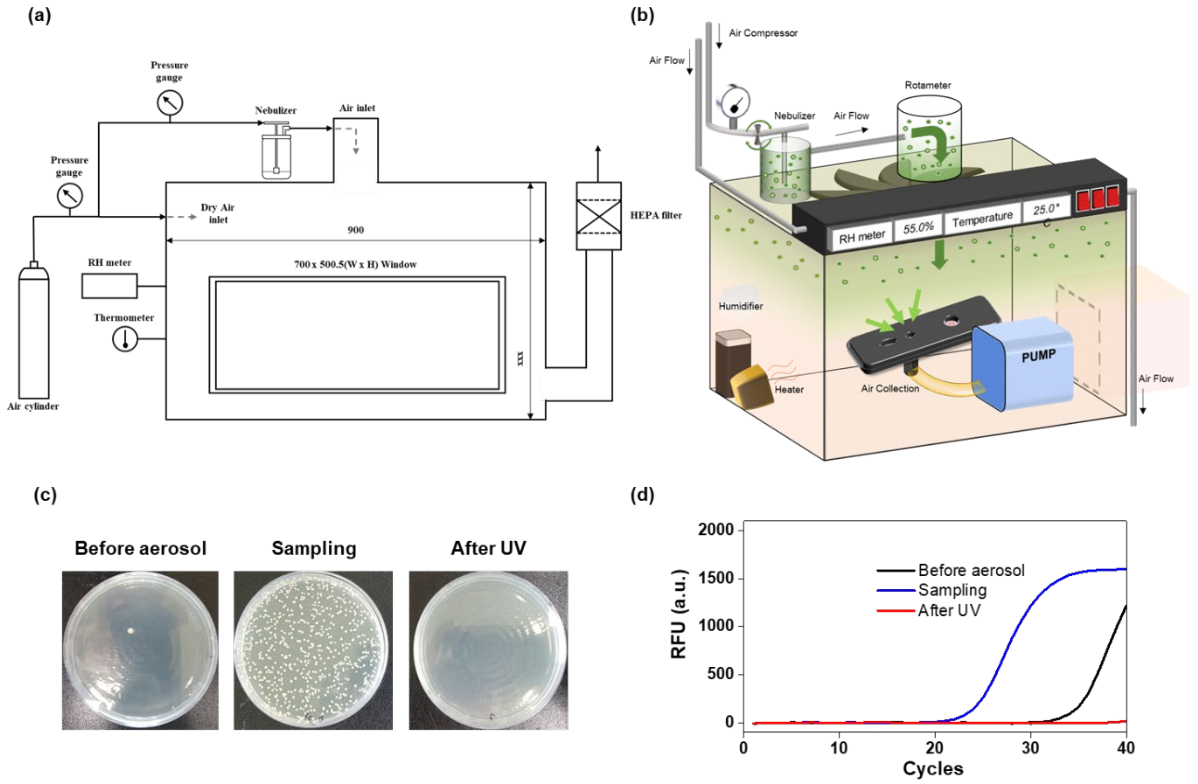

Figure S1. Cabinet system for bacterial aerosol experiments. (**a**) Drawing of the cabinet system, (**b**) schematic of overall cabinet facility, (**c**) bacteria detection result by colony counting using impactor sampler in the cabinet, and (**d**) bacteria detection result by the paper kit and qPCR in the cabinet.

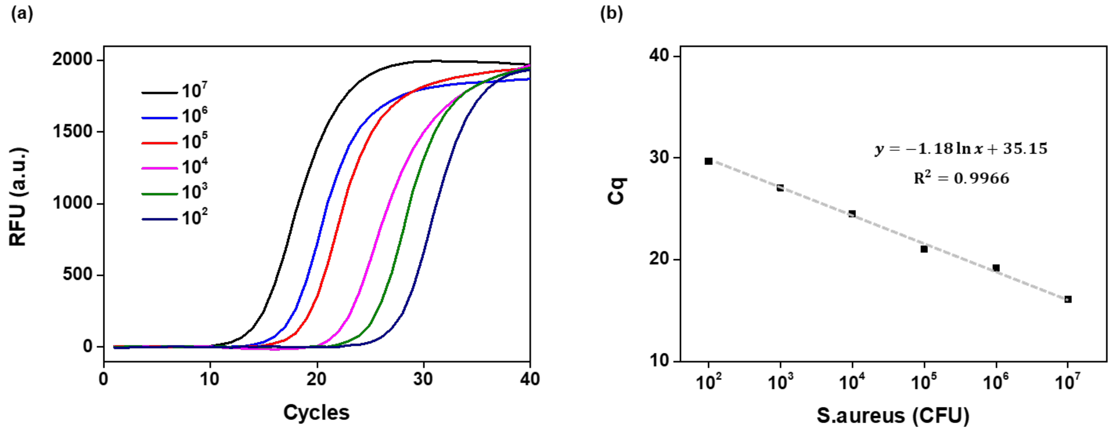

Figure S2. Quantitative real-time PCR (qPCR) analysis. (**a**) Real-time PCR curves of various ranges of *S. aureus* bacteria. (**b**) Linear regression between Cq value and quantity of *S. aureus*.

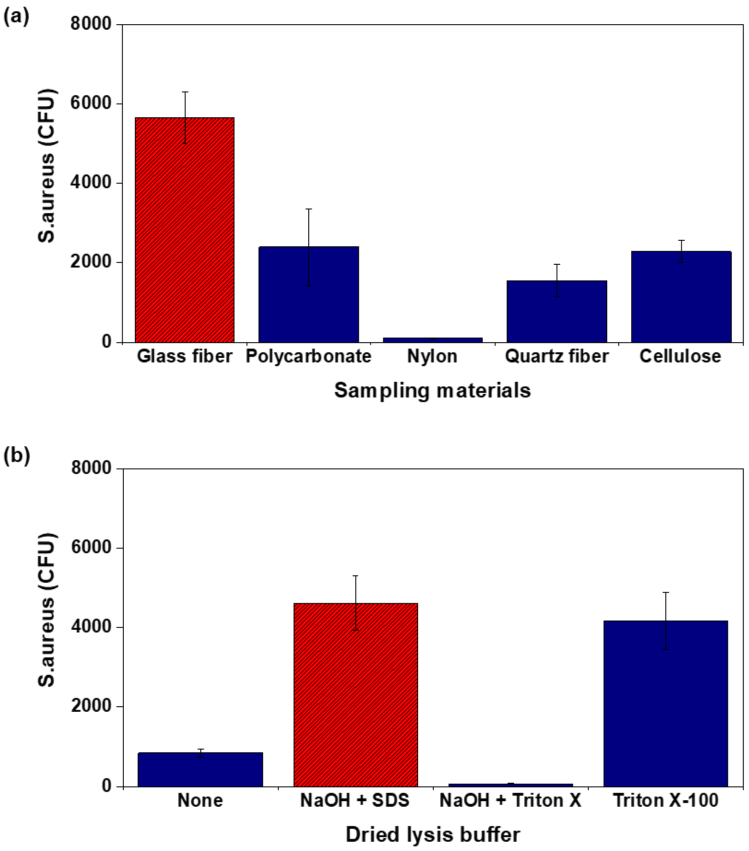

Figure S3. (**a**) Collection results of airborne bacteria on the sampling pad in the kit using various materials. (**b**) Comparison of DNA extraction results for lysis buffer component dried on the sampling pad. All CFU values were measured by qPCR (N = 3).

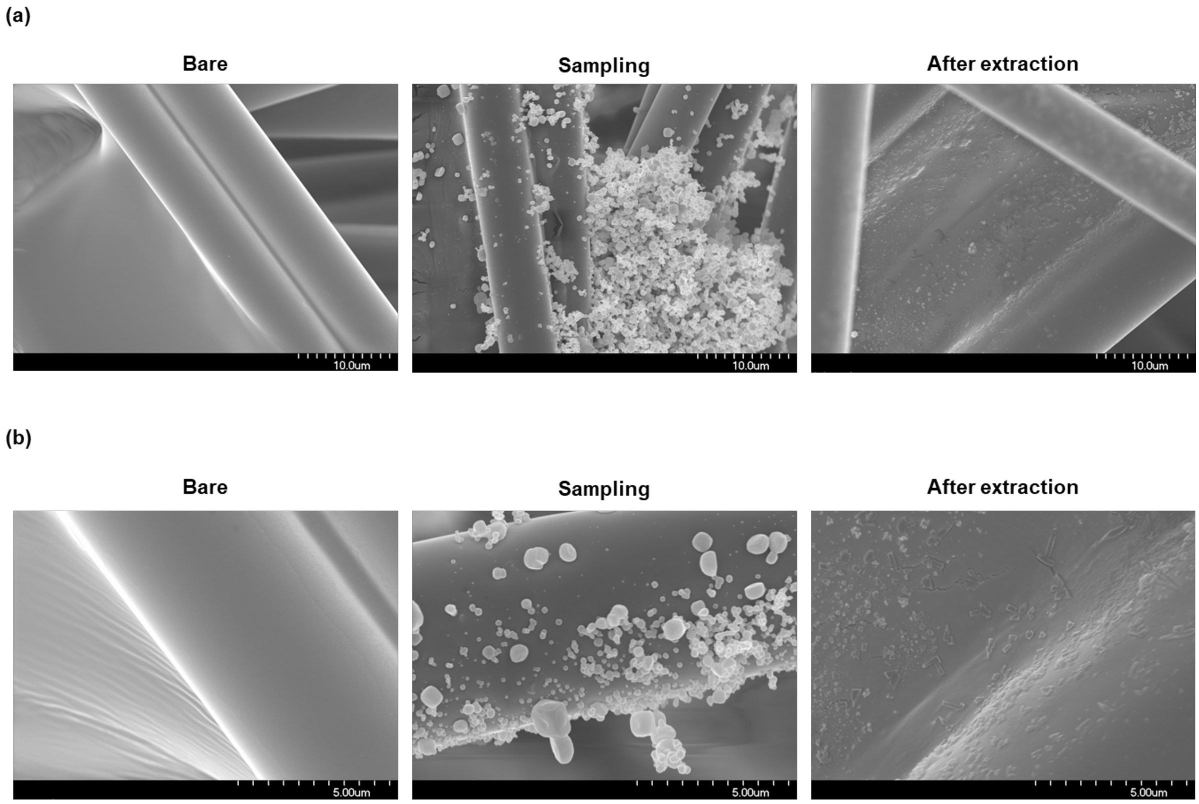

Figure S4. SEM images of sampling pad on the paper kit. Microscopic image of pad surface was captured without any treatment (Bare), for air sampling of 10^4 CFU *S. aureus* aerosolized condition (Sampling), and after whole extraction process (After extraction). (**a**) 3000 times enlarged, and (**b**) 10,000 times enlarged.
